# A pilot study on the use of interferon beta-1a in early Alzheimer’s disease subjects

**DOI:** 10.1186/1742-2094-11-30

**Published:** 2014-02-13

**Authors:** Luigi Maria Edoardo Grimaldi, Giuseppe Zappalà, Francesco Iemolo, Anna Elisa Castellano, Stefano Ruggieri, Giuseppe Bruno, Andrea Paolillo

**Affiliations:** 1 U.O. di Neurologia, Fondazione Istituto San Raffaele “G. Giglio” di Cefalù, Contrada Pietrapollastra, 90015 Cefalù, PA, Italy; 2 U.V.A. Neurologia, Azienda Ospedaliera Garibaldi-Nesima, Via Palermo 135, 95122 Catania, Italy; 3 U.O.C. Provinciale di Neurologia, Via Papa Giovanni XIII, 97019 Vittoria, RG, Italy; 4 Clinica Neurologica, IRCCS Neuromed, Via Atinense, 18, 86077 Pozzilli, IS, Italy; 5 Centro U.V.A., Dipartimento di Scienze Neurologiche, Università degli Studi di Roma “La Sapienza”, Viale dell’Università, 30, 00185 Roma, Italy; 6 MerckSerono S.A., Merck Serono-Italy An affiliate of Merck KGA, Darmsdadt, Germany, Via Casilina 125, 00176 Roma, Italy

**Keywords:** Alzheimer’s disease, clinical trials, randomized, controlled, multiple sclerosis, interferon

## Abstract

Despite the fact that multiple sclerosis (MS) and Alzheimer’s disease (AD) share common neuroimmunological features, interferon beta 1a (IFNβ1a), the well-established treatment for the prevention of disease progression and cognitive decline in MS patients, has never been used in AD. We evaluated the safety and efficacy of IFNβ1a in subjects affected by mild-to-moderate AD in a double-blind, randomized, placebo-controlled, multicenter pilot study. Forty-two early Alzheimer’s patients were randomized to receive either a 22 mcg subcutaneous injection of IFNβ1a or placebo three times per week. A treatment period of 28 weeks was followed by 24 weeks of observation. IFNβ1a was well tolerated and adverse events were infrequent and mild to moderate. Although not statistically significant, a reduction in disease progression during follow-up was measured in IFNβ1a-treated patients by the Alzheimer’s Disease Assessment Scale cognitive subscale. Interestingly, the treatment group showed significant improvements in the Instrumental Activities of Daily Living and Physical Self-maintenance Scale. This study suggests that IFNβ1a is safe and well tolerated in early AD patients, and its possible beneficial role should be further investigated in larger studies.

## Introduction

Alzheimer disease (AD) is one of the major health problems affecting the aging world [1,2]. The disease is characterized by the accumulation in the brain of abnormally folded protein fragments (amyloid beta peptide, Aβ, and tau protein) forming amyloid plaques and neuronal tangles [3] whose appearance is associated with low-grade inflammation, advancing neuronal loss, and progressive cognitive decline. Plaque-associated microglia are able to respond to various stimuli by increasing their expression of histocompatibility complex (MHC) class II antigens and complement receptors as well as proinflammatory cytokines such as IL1β, IL6, and tumor necrosis factor-alpha (TNF-α) [4-6]. In addition, vascular lesions affecting the cerebral endothelium can alter the blood–brain barrier (BBB), especially in AD patients with predisposing vascular abnormalities such as hypertension, cardiovascular disease and diabetes [7]. Similarly, in multiple sclerosis (MS), an immune-mediated disease of the brain characterized by inflammatory-mediated degenerative processes affecting the white matter of the central nervous system and causing demyelination of neuronal axons, microglia and astrocytes are activated and express elevated MHC class II antigen expression, as well as intercellular adhesion molecules (ICAM-1) and proinflammatory cytokines including IL1β, IL6 and TNF-α [8-11].

Interferon beta (IFNβ) was first tested for its antiviral property for the possible involvement of viral infections in the pathogenesis of MS and is now widely used for its immunomodulatory and antiproliferative properties in the treatment of this demyelinating disease [12]. Some of the mechanism of action of IFNβ include a shift from pro- to anti-inflammatory cytokines production [13,14]; inhibition of T-cell activation, blockage of production of oxygen free radicals by mononuclear phagocytes, and reduced expression of MHC II [15]; a protective role against BBB disruption by reducing the activity of metalloproteases and thereby preventing T-cells infiltration in the CNS [16]; a neuroprotective effect on retinal ganglion cells survival and stimulation of the secretion of nerve growth factors by endothelial cells [17,18].

Interestingly, IFNβ1a significantly prevented cognitive decline in a large cohort of patients with MS, thus suggesting that modulation of neuroinflammatory pathways might prevent cognitive decline in humans [19].

At present, no effective treatment is available to stop disease progression in AD. Several immunomodulating strategies in experimental animal models and human clinical trials have recently attempted to alter the immunological pathways underlying the pathological changes seen in AD patients with conflicting results [20,21]. Based on the similarity of some of the immunopathological pathways shared by AD and MS, the similar alteration of the BBB, as well as the positive effects on cognition in MS patients treated with IFNβ1a, we evaluated in a proof-of-concept pilot trial the safety and possible efficacy of subcutaneous (s.c.) low dose IFNβ1a in patients diagnosed with early mild AD.

## Methods

### Study design

This double-blinded, randomized, placebo-controlled, multicenter pilot study (clinicaltrials.gov Identifier: NCT01075763) was conducted according to the declaration of Helsinki. After receiving protocol approval from the Local Ethical Committee, we obtained informed consent from all patients. Patients were considered eligible if they were between 50 and 75 years of age, were diagnosed with AD according to the Diagnostic and Statistical Manual of Mental Disorders, 4th edition (DSM-IV), had a Mini Mental State Examination (MMSE) score between 20 and 26, and were supervised by a caregiver. Exclusion criteria included: use, in the previous 3 months, of statins, nonsteroidal anti-inflammatory drugs, steroids, or cholinesterase inhibitors that could modify the course of the disease; a Modified Hachinski Ischemic Score ≤4; inability to undergo neuropsychological evaluation; significant liver, thyroid or hematological dysfunctions; severe cardiac disease, or epilepsy; a history of depression unresponsive to medication; past medical history of suicidal ideation; current use of hypnotic, anxiolytic, antidepressant, antipsychotic or anticholinergic drugs; and, finally, any known allergic reactions to interferons or other components of the study drug.

A total of 47 patients were screened at 5 AD centers, and 42 patients were enrolled between December 2004 and May 2007. Patients’ baseline characteristics are detailed in Tables 1 and 2.

**Table 1:** Adverse events recorded in Placebo-and IFNβ 1-treated subjects

**Adverse event**	**Placebo**		**IFN β 1a**		**Total**
**Severity**	**Mild**	**Moderate**	**Mild**	**Moderate**	
Articular pain	2^a^	0	0	0	2
Fever	0	0	1^a^	0	1
High cholesterol	0	0	3^c^	0	3
High level of ALT and AST	0	1^b^	0	1^b^	2
High pseudocholinesterase	1^c^	0	1^c^	1^c^	3
High triglycerides	0	0	0	1^c^	1
High TSH	0	1^c^	0	0	1
Low blood pressure	1^c^	0	0	0	1
Repolarization ventricular anomaly	0	0	1^c^	0	1
Right ear buzzing	1^b^	0	0	0	1
Flu-like syndrome	1^c^	0	0	0	1
TOTAL	6	2	6	3	17

The number of adverse events in the two treatment arms is reported in details subdivided by severity degree. The relation to drug administration is shown as follows: ^a^probably related; ^b^unlikely related; ^c^unrelated. ALT, alanine aminotransferase; AST, aspartate aminotransferase; TSH, thyroid stimulating hormone.

**Table 2:** Demographics and physical characteristics at baseline

**Characteristic**	**Placebo**^**a**^	**Interferon beta 1a**^**a**^	***P *** **value**
Age at informed consent (yrs)	64.57 ± 6.4	63.0 ± 9.07	0.57
Height (cm)	165.11 ± 7.52	163.0 ± 9.01	0.42
Weight (kg)	71.59 ± 9.92	68.17 ± 10.04	0.27
Systolic pressure (mmHg)	131.05 ± 7.56	164.74 ± 18.26	0.31
Diastolic pressure (mmHg)	79.47 ± 6.64	116.26 ± 19.2.	0.26
Pulse rate (bpm)	70.94 ± 11.88	71.56 ± 6.97	0.86
Sex^b^	8 (male); 11 (female)	8 (male); 15 (female)	0.62
Concomitant age-related diseases different from AD	14 (yes); 4 (no)	14 (yes); 8 (no)	0.94

^a^Values are expressed as means ± SD. ^b^Sex values are reported as absolute values.

**Figure 1:**
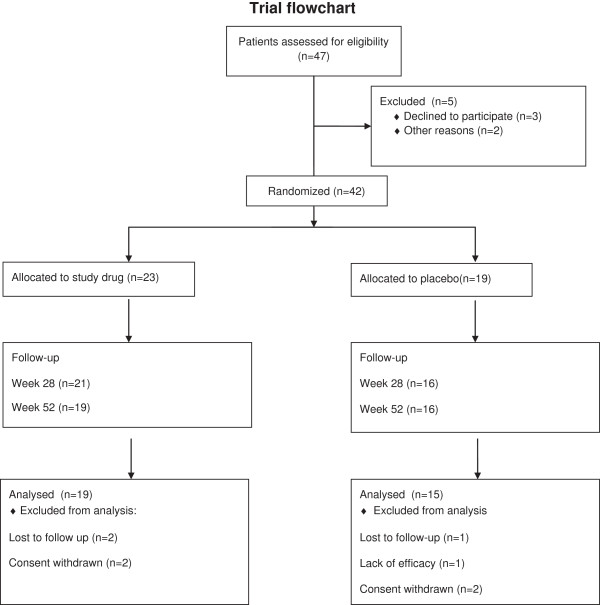
Trial flowchart giving details on the number of patients screened and taking part in each phase of the trial.

Subjects were randomized into two groups: one (n = 23) receiving IFNβ1a (Rebif®, Merck Serono S.A. - Geneva, Switzerland), 22 mcg subcutaneous injection (s.c.), three times per week; the other (n = 19) receiving placebo with the same schedule and route of administration. The treatment period was 28 weeks and patients were followed for up to 52 weeks (Figure 1). Outcome measures were monitored at baseline, week 28, and week 52. The primary efficacy evaluation was the Alzheimer’s Disease Assessment Scale cognitive subscale (ADAS-Cog). Secondary efficacy evaluations included Global Deterioration Scale (Global DS), Clinician Interview Based Impression of Change (CIBIC-plus), Mini-Mental State Examination (MMSE), ADAS non-cognitive subscale (ADAS-NonCog), Instrumental Activities of Daily Living (IADL), Physical Self-Maintenance Scale (PSMS), and Geriatric Depression Scale (GDS). At each routine visit serum chemistry, hematological, and urine abnormalities were evaluated, and physical and neurological examinations were performed.

### Statistical analysis

Primary and secondary endpoints were assessed by arm-descriptive analysis (mean, median, SD, range) of measured values at each time point and related changes over time. In addition, a preliminary assessment of normality was performed for each continuous scale. Mean differences with screening/baseline was tested using repeated measures T-test and ANOVA, as appropriate. The other secondary outcome measures were assessed at screening/baseline and at week 12, week 28 and week 52 (Mini Mental Status Examination (MMSE), ADAS, non-cognitive subscale (ADAS-NonCog) or at week 28 and 52 (Instrumental Activities of Daily Living (IADL), Physical Self-Maintenance Scale (PSMS), Clinician’s Interview Based Impression of Change (CIBIC-PLUS), Geriatric depression scale (GDS), and Global Deterioration Scale). Mean differences between follow-up and baseline of nonparametrically distributed variables (all test with the exception of GDS) were assessed using Wilcoxon scores and the Kruskal-Wallis test, while arm-specific differences were analyzed by Wilcoxon signed-rank test. Qualitative variables (for example, CIBIC-PLUS) were compared as proportions and tested using chi-square or Fischer exact tests, as appropriate.

## Results

### Safety

The study drug was well tolerated and adverse events were reported in 4/42 (9 %) of the subjects, with no significant differences in the two arms. None of the adverse events were serious or resulted in death or early termination of study. The total number of adverse events was 17, 12 of which were mild and 5 were moderate. Moreover, only three of the adverse events were related to drug administration (Table 1). Ten events had resolved by the end of the study.

No significant changes in vital signs, or hematological or urinalysis parameters were reported; although, a mild decrease of cholesterol levels was observed in the IFNβ1a group but not in the placebo arm (3/19 versus 0/15, *P* = < 0.05).

### Efficacy

Patients in the placebo and study drug randomized groups shared the same baseline characteristics with no significant differences, as shown in Table 2. Similarly, the baseline means scores of all parameters measured in the study did not differ in the two arms, confirming that the randomization did not generate any bias in the protocol, as detailed in Table 3.

**Table 3:** Neuropsychological tests values at baseline

**Test**	**Placebo**^**a**^	**Interferon beta 1a**^**a**^	***P *** **value**
ADAS-Cog^b^	19.81 ± 6.14	18.34 ± 7.85	0.34
MMSE^c^	22.93 ± 1.78	23.45 ± 2.13	0.41
ADAS-NonCog^d^	6.06 ± 5.0	4.36 ± 4.12	0.30
PSMS^e^	6.4 ± 0.91	6.42 ± 1.01	0.98
GDS^f^	10.66 ± 6.53	10.10 ± 6.12	0.79
Global DS^g^	2.86 ± 0.74	3.0 ± 0.47	0.50
IADL^h^	6.2 ± 1.56	6.1 ± 1.44	0.68

^a^Values are expressed as means ± SD. ^b^ADAS-cog, Alzheimer’s Disease Assessment Scale-cognitive subscale; ^c^MMSE, Mini Mental State Examination; ^d^ADAS-Non-Cog, Alzheimer’s Disease Assessment Scale-Non-Cognitive Section; ^e^PSMS, Physical Self-Maintenance Scale; ^f^GDS, Geriatric Depression Scale; Global, ^g^Global Deterioration Scale (DS) for Assessment of Primary Degenerative Dementia; ^h^IADL, Instrumental Activities of Daily Living Scale.

The primary outcome measure, ADAS-Cog, showed no significant progression of AD during the 28-week treatment period, while a worsening was observed during follow-up, as shown by the delta values of ADAS-Cog score when comparing the different time points of the trial (Table 4). However, no significant differences of overall efficacy were observed between placebo and treatment group.

**Table 4:** **Analysis of change (delta values) of outcomes at study time points**^a^

**Test**	**Treatment**	**Delta value T1**^**b **^**versus T2**^**c**^	**Delta value T2 versus T3**^**d**^	**Delta value T1 versus T3**
**ADAS-Cog**^**e**^	IFNβ 1a	0.144 ± 1.16	**−2.17 ± 1.01** (p = 0.031)	−2.02 ± 1.58
	Placebo	2.25 ± 1.05	−2.78 ± .1.02	−0.52 ± 1.86
**MMSE**^**f**^	IFNβ 1a	0.78 ± 0.52	**−1.81 ± 0.69** (p = 0.037)	−1.02 ± 1.03
	Placebo	0.73 ± 0.98	−1.68 ± 0.76	−0.95 ± 1.0
**ADAS-NonCog**^**g**^	IFNβ 1a	−0.26 ± 0.53	−0.84 ± 0.35	−1.10 ± 0.65
	Placebo	0.86 ± 0.75	−2.0 ± 1.099	−1.13 ± 1.26
**PSMS**^**h**^	IFNβ 1a	0.31 ± 0.23	**1.05 ± 0.66** (p = 0.0459)	**1.36 ± 0.76** (p = 0.025)
	Placebo	0.6 ± 0.6	−0.13 ± 0.29	0.46 ± 0.33
**GDS**^**i**^	IFNβ 1a	0.42 ± 0.94	−1.10 ± 0.79	−0.68 ± 1.35
	Placebo	2.0 ± 1.34	**1.57 ± 0.83** (p = 0.0418)	**3.57 ± 1.24** (p = 0.009)
**Global DS**^**j**^	IFNβ 1a	−0.05 ± 0.17	−0.21 ± 0.12	−0.26 ± 0.18
	Placebo	−0.13 ± 0.13	−0.2 ± 0.10	−0.33 ± 0.15
**IADL**^**k**^	IFNβ 1a	0.26 ± 0.34	**0.94 ± 0.37** (p = 0.0306)	**1.20 ± 0.59** (p = 0.041)
	Placebo	0.6 ± 0.36	−0.06 ± 0.18	0.53 ± 0.36

^a^Values are shown as means ± SEM; Negative change indicates worsening of condition. In brackets are reported p values (by arm) when statistically significant. ^b^T1 = baseline (week 0); ^c^T2 = end of treatment (week 28); ^d^T3 = follow-up (week 54). ^e^ADAS-cog, Alzheimer’s Disease Assessment Scale-cognitive subscale; ^f^MMSE, Mini Mental State Examination; ^g^ADAS-Non-Cog, Alzheimer’s Disease Assessment Scale-Non-Cognitive Section; ^h^PSMS, Physical Self-Maintenance Scale; ^i^GDS, Geriatric Depression Scale; Global, ^j^Global Deterioration Scale (DS) for Assessment of Primary Degenerative Dementia; ^k^IADL, Instrumental Activities of Daily Living Scale.

The same trend was observed for MMSE, and ADAS-NonCog scores, thus indicating a positive effect of treatment on the disease decline but without significant differences between the two treatments arms (Table 4).

Interestingly, there was a significant improvement of both PSMS and IADL scores in the IFNβ1a group at follow-up compared with both baseline and end of treatment. The Global Deterioration Scale showed a modest decline in patients of the two arms during the treatment period and between baseline and follow-up. However, no significant differences were observed. Similarly, CIBIC-plus, measured at 28 and 52 weeks, did not show any differences between arms (data not shown). Conversely, the GDS score significantly improved in the placebo group, overall and during follow up (Table 4).

## Discussion

New treatments for MS are expected to become available within the next 2 years [22]. Conversely, AD is still lacking a convincing strategy to halt the progressive decline of cognitive function associated with protein accumulation within the brain. The now well-established contribution of inflammatory mechanisms to the pathogenesis of plaque formation offers an interesting target for treatment. At present, numerous attempts to alter the inflammatory processes in AD have been performed using different anti-inflammatory drugs [20,21].

Although IFNβ1a has a well-established positive safety profile in the young adult (the typical MS population), little is known in older patients (typical AD population). We assessed the safety of IFNβ1a for the treatment of patients affected by mild AD and found that this drug was well tolerated. The number of adverse events reported was low and their severity was mild to moderate and comparable between the two arms of the study.

Our study was clearly not powered to demonstrate efficacy. However, although no statistically significant difference between the IFNβ1a and the placebo groups was reported for the major outcome measure of the study, the ADAS-Cog score, the improvement noted in the IFNβ1a treatment group was maintained during the 24-week follow-up period. Similarly, improvement in the ADAS-NonCog score was maintained. Importantly, significant improvements in the IADL and in the PSMS scores, extending to the follow-up phase, were observed in the IFNβ1a treatment group.

These results show that, although treatment with IFNβ1a did not perform better than placebo during the treatment period, a slower decline could be observed in the active drug group during the follow-up period, indicating a prolonged protective effect of IFNβ1a.

Paradoxically, but not surprisingly, we found that the placebo had an antidepressant effect, as shown by the significant improvement in the Geriatric Depression Scale, suggesting that this effect may have been responsible for improvement in the placebo group during the 6 months of treatment. Mood elevation engendered by the placebo may have counterbalanced any positive cognitive outcome of the active drug, which is well known to produce a transient mood decline during the first months of treatment [23].

Interestingly, a cholesterol lowering trend was observed in IFNβ1a treated patients, indicating a possible additional mechanism for its efficacy since cholesterol might play a role in the pathogenesis of AD, and cholesterol lowering agents, such as statins, have been tested as treatment for AD [24,25]. Recently, Weinstock-Gutmann *et al*. [26], reported that in early MS patients, higher levels of low density lipoprotein cholesterol (LDL-C) and total cholesterol (TC) are associated with increased inflammation (by MRI evaluation) in subjects affected by a clinically isolated syndrome and treated with intramuscular injection of IFNβ1a.

In conclusion, our study demonstrates the tolerability, short-term safety and feasibility of using IFNβ1a in treatment of older patients at risk for AD-related cognitive decline. Our findings suggest that the strategy used here should be further investigated in larger studies targeting subjects in early stage disease where inflammatory-mediated facilitation of misfolded-protein accumulation is more likely to occur.

## Abbreviations

ADAS-cog: Alzheimer’s Disease Assessment Scale-cognitive subscale; ADAS-Non-Cog: Alzheimer’s Disease Assessment Scale-Non-Cognitive Section; ALT: alanine aminotransferase; AST: aspartate aminotransferase; BBB: blood–brain barrier; GDS: Geriatric Depression Scale; Global: Global Deterioration Scale for Assessment of Primary Degenerative Dementia; IADL: Instrumental Activities of Daily Living Scale; ICAM-1: intercellular adhesion molecules; IFNβ1a: interferon beta 1a; LDL-C: low density lipoprotein cholesterol; MHC: major histocompatibility complex; MMSE: Mini Mental State Examination; PSMS: Physical Self-Maintenance Scale; TSH: thyroid stimulating hormone.

## Competing interests

The authors declare that they have no competing interests.

## Authors’ contributions

LMEG generated the idea at the base of the study, searched for economical support, organized and carried out the study at its Center, wrote and edited the final version of the manuscript; GZ enrolled the majority of subjects and drafted the first version of the manuscript, FI, AEC, GB and SR organized and carried out the study at their Centers, collected and revised the clinical data and contributed to the preparation of the manuscript; AP contributed to the overall development and organization of the study, collected and analyzed the data and drafted and corrected the manuscript. All authors read and approved the final manuscript.
